# Evaluation of Social and Clinical Factors Associated with Adverse Drug Reactions Among Children with Drug-Resistant Tuberculosis in Pakistan

**DOI:** 10.3390/tropicalmed10070176

**Published:** 2025-06-20

**Authors:** Muhammad Soaib Said, Razia Fatima, Rabbiya Ahmad, Mahmood Basil A. Al Rawi, Faheem Jan, Sobia Faisal, Irfanullah Khan, Amer Hayat Khan

**Affiliations:** 1 Discipline of Clinical Pharmacy, School of Pharmaceutical Sciences, University Sains Malaysia, Gelugor 11700, Malaysia; rabbiyahamd@gmail.com (R.A.); malrawi@ksu.edu.sa (M.B.A.A.R.); 2 The Common Management Unit for TB HIV AIDS and Malaria, Ministry of National Health Services, Regulation and Coordination, Islamabad 44000, Pakistan; drraziafatima@gmail.com (R.F.); drfaheem82@gmail.com (F.J.); drsobia@ntp.gov.pk (S.F.); 3 Department of Optometry, College of Applied Medical Sciences, King Saud University, Riyadh 11451, Saudi Arabia; 4 Department of Integrative Biology, Oklahoma State University, 420 Life Sciences West, Stillwater, OK 74078, USA; irfanullah148@yahoo.com

**Keywords:** adverse drug reactions, children, risk factors

## Abstract

(1) Background: The occurrence, intensity, and characteristics of adverse drug reactions (ADRs) caused by anti-tuberculosis (TB) drugs have consistently been a subject of worry. There is a lack of published research from Pakistan regarding the negative effects of anti-TB treatment on children, specifically about ADRs. In this study, we aimed to investigate the ADR associated with anti-DR-TB treatment in children. (2) Methods: A prospective longitudinal study was conducted in the multicenter setting of Khyber Pakhtunkhwa, Pakistan. A total of 450 TB children in multicenter hospitals under ATT were assessed for ADRs. Naranjo Causality Assessment and Hartwig’s Severity Assessment Scale were used to evaluate the causality and severity. (3) Results: A total of 300 (66.66%) ADRs were reported in 450 people with DRTB. Anemia was the most frequently observed ADR (37.6%) followed by nausea and vomiting (18.6%). On multivariate analysis, the independent variables that had a statistically significant positive association with ADRs were participants aged, 5–14 years (AOR, 0.3 (0.1–0.5), *p* ≤ 0.001), normal weight (1.1 (2.0–1.9), *p* < 0.001), and children having comorbidities (AOR, 0.5 (0.1–0.8), *p* ≤ 0.001). (4) Conclusions: Our findings advocate for personalized treatment approaches, incorporating nutritional support, comprehensive comorbidity management, and vigilant monitoring to mitigate ADRs and improve treatment outcomes.

## 1. Introduction

The occurrence, intensity, and characteristics of adverse drug reactions (ADRs) caused by anti-tuberculosis (TB) drugs have always been a subject of concern [1]. Research has demonstrated that the use of pharmacological treatments can lead to unwanted ADRs, including joint pain, allergic responses, liver toxicity, neurological issues, and gastrointestinal difficulties [2]. ADRs associated with anti-TB treatment (ATT) have consistently been a significant issue because of the increasing expenses of treatment, the need for frequent visits to health facilities, and the potential for hospitalizations in severe cases [3]. ADRs can lead to treatment interruption, which may contribute to treatment failure, the development of drug resistance, and, ultimately, an increase in TB cases and, in some instances, fatalities [4]. Among the estimated 25,000 to 32,000 children who develop DR TB every year [5]. The majority of TB cases in children are found in low-income and lower-middle-income countries that have inadequate resources and poorly developed procedures for reporting ADRs associated with treatment [6].

Prolonged exposure to ATT elevates the likelihood of ADRs [7]. These ADRs can be both mild and potentially fatal. An ADR that is severe enough to require withdrawal of one of the main anti-TB medications can lead to various consequences, including higher rates of illness and death [8]. Consequently, there is an increased likelihood of treatment failure and relapses. Understanding the risk categories is crucial as it can reduce both the cost and the likelihood of severe drug-related ADRs. Past studies have found a strong correlation between ADRs and certain characteristics. These factors include being female, having a history of past ADRs, drinking, HIV co-infection, genetic factors, dietary deficiencies, smoking, having a history of previous TB treatment, and having other medical conditions [9,10,11]. ADRs are a significant cause of illness and death [12]. Therefore, it is crucial to identify ADRs and establish a causal connection between the medication and the negative occurrence. Causality assessment of ADRs is a technique employed to determine the degree of association between drug exposure and the incidence of ADRs [13]. On the other hand, the severity of ADRs is determined by the impact they have on the everyday lives of people with DRTB [14]. The incidence, severity, and characteristics of ADRs caused by ATT have consistently been a cause for worry [15]. The objective of this study was to examine the occurrence of ADRs, factors that contribute to the risk of ADRs, and the impact of anti-DR treatment on children with TB.

## 2. Materials and Methods

### 2.1. Study Design

The children aged 16 and under participated in a prospective longitudinal study conducted at two Directly Observed Treatment (DOTS) centers in Pakistan. The data were collected from the Tuberculosis Clinic, Peshawar, Pakistan (Regional Programmatic Drug Resistant TB Center, LRH) and the Tuberculosis Clinic, Abbottabad, Pakistan (Regional Programmatic Drug-resistant TB Center, Ayub Medical Complex).

ADRs of anti-DR-TB were evaluated for a total of 450 children who were registered for DRTB therapy from 1 January 2013 to 1 June 2024. Every month, people with DR-TB were checked for the prevalence of ADRs, and parents of young people with DR-TB were advised to contact the clinician at any moment if they had any concerns. Their laboratory reports and medical files were assessed to prescribe auxiliary medications to assess and manage ADRs in the research population. The severity of ADRs was determined by laboratory reports on hematologic disorders, symptomatic events reported by caregivers, and physical examinations by pediatricians and clinicians (if laboratory reports were unable to identify ADRs. The Naranjo and Hartwig scale was used to determine the causation and severity of ADRs in research participants [16].

### 2.2. Study Population

All the children with DR-TB were enrolled. All confirmed children with DR-TB, regardless of gender and presence of co-morbidity, will be under consideration.

### 2.3. Inclusion and Exclusion Criteria

The inclusion criteria were children 16 years and below diagnosed with DR-TB (multi-drug-resistant tuberculosis (MDR-TB) and Rifampicin-Resistant tuberculosis (RR-TB). Molecular diagnostics were used for the initial detection of DR-TB, specifically, Xpert MTB/RIF (Cepheid, Sunnyvale, CA, USA). In cases with RR-TB detected, additional first-line DST was performed using line probe assays (LPA) by FluoroType^®^ MTB (Hain Lifescience GmbH, Nehren, Germany) to confirm isoniazid resistance, enabling classification as MDR-TB where applicable. The exclusion criteria were death or loss to follow-up (LTFU) within the first 15 days of ATT (early death or early LTFU, avoiding data capture). Cancer patients and people living with HIV were excluded.

### 2.4. Treatment Regimen

All patients received standardized WHO-recommended second-line treatment regimens based on their resistance profiles. For children with confirmed RR-TB, a shorter regimen was used where appropriate, while children with confirmed MDR-TB (rifampicin + isoniazid resistance) received an individualized longer regimen.

Children in this study received treatment regimens aligned with WHO-recommended protocols for drug-resistant tuberculosis (DR-TB), adjusted according to individual drug susceptibility profiles. The typical regimen included a second-line injectable agent, Amikacin, administered during the intensive phase, depending on the patient’s age and baseline audiometry results. A fluoroquinolone—either levofloxacin or moxifloxacin—was included as a key component of the regimen. Other core agents commonly used were ethionamide, cycloserine, linezolid, and clofazimine. Pyrazinamide and ethambutol were added as companion drugs when drug susceptibility testing (DST) indicated sensitivity.

### 2.5. Duration of Treatment

Short regimen (for eligible RR-TB): 9–11 months. Longer regimen (for MDR-TB or extensive resistance): 18–24 months. Treatment duration was adjusted based on clinical response and drug susceptibility.

### 2.6. Treatment Setting

Most patients were treated in a mixed setting: Initial hospitalization was provided during the intensive phase (typically 4–8 weeks) for close monitoring, especially for ADR detection and management. This was followed by an ambulatory phase, where patients continued treatment under directly observed therapy (DOT) in outpatient or home-based settings.

### 2.7. Ethics Statement

Ethical approval was granted by the Research and Ethics Committee CMU, Ministry of National Health Services, Regulation and Coordination, Islamabad, Pakistan vide notification no. F.No.27-IRB-CMU-2023. 

### 2.8. Data Analysis

The data analysis tool utilized was IBM SPSS Statistics 27. Throughout the data-gathering process, all extracted information was coded into variables, and cases were entered using their serial numbers. ADR determinants were assessed with the application of logistic regression analysis. In the multivariate analysis, every variable that was significant in the univariate analysis was included. For every independent variable, odds ratios (ORs) and their 95% confidence intervals (CIs) were calculated. It was deemed statistically significant when *p* < 0.05.

## 3. Results

### 3.1. Sociodemographic Characteristics

The study analyzed 450 individuals, 300 (66.66%) experiencing ADRs and 150 not experiencing ADRs. Age was significantly associated with ADRs (*p* = 0.001), with the highest proportion of ADRs in the 5–14 years age group. Weight also showed a significant difference (*p* = 0.002), as underweight individuals experienced more ADRs than those of normal weight. Retreated cases (*p* = 0.002) and the presence of comorbidities (*p* = 0.005) were other factors significantly associated with ADRs. Comorbidities included in the study were diabetes, anemia, chronic respiratory conditions, and liver function abnormalities. The results are discussed in Table 1.

### 3.2. Treatment Outcomes

The study presents treatment outcomes for a cohort of 450 individuals. A substantial majority, 336 people with DRTB (74.7%), were classified as cured, reflecting a high success rate for the treatment regimen used. Additionally, 20 people with DRTB (4.4%) completed the treatment, suggesting that nearly 80% of the cohort either achieved a cure or completed treatment. Conversely, 20 participants (4.4%) experienced treatment failure, indicating a need for alternative strategies or interventions for a subset of participants. A total of 25 participants (5.6%) were lost to follow-up, which underscores the importance of strategies to improve patient retention and adherence to treatment protocols. Mortality was observed in 24 participants (5.3%), highlighting the severity of the condition and the critical need for continuous improvements in treatment approaches and supportive care. All these results are explained in Table 2.

### 3.3. Onset Time of ADRs of ATT

In Table 3, the study examines the onset time and prevalence of ADRs among 300 participants undergoing treatment, revealing critical insights into the temporal patterns and frequency of various ADRs.

Nausea and vomiting emerged as the most common ADR, reported by 55 participants (18.6%) with a median onset time of 22 days (IQR: 6–34 days). Abdominal pain was noted in 47 participants (15.6%), typically manifesting at a median of 62 days (IQR: 24–98 days). Allergic reactions were reported by 31 participants (10.3%), with a median onset of 12 days (IQR: 6–21 days). Notably, anemia was the most prevalent ADR, affecting 113 participants (37.6%), with a median onset of 15 days (IQR: 6–31 days).

### 3.4. Naranjo Causality Assessment Scale

This study analyzed the types of ADRs in TB treatment, categorizing them as doubtful, possible, probable, or definite. Nausea/vomiting was mostly classified as definite (54.6%), while abdominal pain was most often possible (48.9%). Anorexia showed a balanced distribution across all categories. Arthralgia was predominantly probable (66.66%). Hepatotoxicity was mainly definite (47.3%), and allergy reactions were overwhelmingly definite (67.9%). Anemia, a common ADR, was frequently classified as definite (48.7%). These results highlight the varying certainty levels of ADRs, emphasizing the need for careful monitoring and management during TB treatment. The results are explained in Table 4.

### 3.5. Hartwig Severity Assessment Scale

In this study of ADRs among TB participants, the severity of ADRs was categorized as mild, moderate, and severe. The Hartwig scale categorizes ADRs into mild, moderate, or severe based on clinical outcomes such as the need for treatment modification, hospitalization, or permanent drug discontinuation. We chose the Hartwig scale because of its objective criteria, ease of application in retrospective and prospective settings, and its ability to reflect the clinical impact of ADRs in a structured manner. The majority of ADRs were moderate, accounting for 47% of cases. Mild ADRs constituted 30.7%, while severe ADRs made up 22.3%. These findings underscore the importance of monitoring ADR severity in TB treatment, as nearly half of the participants experienced moderate reactions, and a significant portion faced severe ADRs, necessitating careful management to ensure patient safety and treatment efficacy. The results are explained in Table 5.

### 3.6. Factors Associated with ADRs

In Table 6, we analyzed the association between various characteristics and the occurrence of ADRs. Gender showed no significant association with ADRs (*p* = 0.64). Age group 5–14 years had a higher likelihood of ADRs (OR 1.3, *p* = 0.05), whereas the 15–16 years group had a lower likelihood (OR 0.3, *p* = 0.001). Underweight participants were more prone to ADRs compared to those with normal weight (OR 0.3, *p* < 0.001). Comorbidities significantly increased the likelihood of ADRs (OR 0.3, *p* < 0.001).

### 3.7. Factors Associated with ADRs (Multivariate Logistic Regression)

In Table 7, we analyzed the association between various characteristics and the occurrence of ADRs. Gender showed no significant association with ADRs (*p* = 0.64). Age group 5–14 years had a higher likelihood of ADRs (OR 1.3, *p* = 0.05), whereas the 15–16 years group had a lower likelihood (OR 0.3, *p* = 0.001). Underweight participants were more prone to ADRs compared to those with normal weight (OR 0.3, *p* < 0.001). Comorbidities significantly increased the likelihood of ADRs (OR 0.3, *p* < 0.001).

## 4. Discussion

The present study provides a comprehensive analysis of ADRs in MDR-TB treatment, underscoring significant sociodemographic and clinical risk factors. The above results showed that 66.66% of the DR-TB participants suffered from ADRs. In comparing our findings to international data on ADRs among children undergoing DR-TB treatment, a more appropriate reference is the study conducted in Tashkent, Uzbekistan, which specifically focused on the pediatric population. This study reported a high burden of ADRs in children with DR-TB, particularly gastrointestinal disturbances, hepatotoxicity, and anemia, which is consistent with the trends observed in our cohort. These parallels reinforce the global challenges of managing complex DR-TB regimens in pediatric populations and the critical need for age-appropriate drug formulations, toxicity monitoring, and safer treatment protocols [17]. The incidence is comparatively high, and if these ADRs are not effectively treated, the impacted individuals may become lost to follow-up, which could result in TB that is extensively resistant to drugs (XDR-TB).

The study analyzed 450 individuals, out of which 300 (66.66%) experienced ADRs. Our analysis indicates that participants aged 5–14 years have a higher likelihood of experiencing ADRs compared to other age groups (OR = 1.3, *p* = 0.05). Conversely, children aged 15–16 years show a reduced risk (OR = 0.3, *p* = 0.001). This variability suggests that younger children may have different metabolic responses to anti-TB medications, potentially due to developmental pharmacokinetics and pharmacodynamics differences. Previous studies corroborate this finding, highlighting that pediatric populations, particularly those in early childhood, exhibit distinct ADR profiles due to immature drug metabolism pathways [18,19].

Weight status emerged as a significant determinant of ADR occurrence. Underweight participants demonstrated a higher predisposition to ADRs, whereas normal-weight individuals had significantly lower odds (OR = 0.3, *p* < 0.001). Malnutrition and underweight status can exacerbate drug toxicity and alter pharmacokinetics, leading to increased susceptibility to ADRs [20]. This underscores the need for nutritional assessments and interventions as integral components of TB treatment regimens, particularly in settings with a high prevalence of malnutrition.

Participants with bilateral lung cavitation were less likely to experience ADRs compared to those with unilateral cavitation (OR = 0.2, *p* = 0.005). The presence of cavitary lesions in TB has been linked to higher bacillary load and more aggressive disease, which may influence drug absorption and metabolism [21]. The differential impact of cavitation on ADRs warrants further investigation to elucidate the underlying mechanisms and optimize therapeutic approaches.

The presence of comorbidities significantly increased the likelihood of ADRs (OR = 0.3, *p* < 0.001). Comorbid conditions, such as HIV, diabetes, and chronic liver diseases, can complicate TB treatment and elevate the risk of drug interactions and toxicity [22]. Our findings highlight the importance of comprehensive comorbidity management and tailored therapeutic strategies to mitigate ADRs in people with DR-TB with coexisting conditions.

The study demonstrates a high cure rate of 74.7%, with an additional 4.4% completing treatment. However, treatment failure (4.4%), loss to follow-up (5.6%), and mortality (5.3%) remain critical concerns. These outcomes align with global TB treatment challenges, emphasizing the need for robust patient support systems to enhance adherence and reduce mortality [11].

The onset and severity of ADRs varied, with nausea and vomiting (18.6%) and anemia (37.6%) being the most prevalent. The timing of ADR onset, ranging from 12 to 62 days, highlights the need for continuous monitoring throughout the treatment course. Moderate to severe ADRs accounted for a significant proportion (47% and 22.3%, respectively), necessitating proactive management to ensure patient safety and treatment efficacy.

Our study categorizes ADRs into doubtful, possible, probable, and definite. Nausea and vomiting were predominantly classified as definite (54.6%), consistent with findings from Chhetri et al., 2008, who reported a high incidence of gastrointestinal side effects in people with DR-TB [23]. Abdominal pain was mostly possible (48.9%), similar to reports by Chung et al., 2022, which highlighted abdominal discomfort as a common but less certain ADR [24].

Anorexia showed a balanced distribution across all causality categories, indicating varying certainty levels in previous studies [25,26]. Arthralgia was predominantly probable (66.66%), aligning with the findings, which documented joint pain as a probable ADR in TB treatment [8].

Allergic reactions were overwhelmingly definite (67.9%), corroborating studies by Forget and Menzies, which emphasized the high certainty of allergic ADRs in TB therapy [27]. Anemia, frequently classified as definite (48.7%), is consistent with the findings of Bahi and colleagues, who noted hematologic ADRs as definite in a significant number of TB cases [28].

The severity assessment using the Hartwig scale revealed that moderate ADRs were most common (47%), followed by mild (30.7%) and severe ADRs (22.3%). This distribution is similar to the study by Massud A and colleagues, which also found that moderate ADRs were most prevalent among people with DR-TB, which also found that moderate ADRs were most prevalent among people with DR-TB [29]. The proportion of severe ADRs in our study (22.3%) is notable and emphasizes the need for vigilant monitoring, as echoed by Singh et al., 2023, who stressed the clinical significance of severe ADRs in pediatric TB treatment [30].

The presence of comorbidities, though not extensively detailed in this study, emerged as a significant risk factor for ADRs. Among those documented, malnutrition, anemia, and previous episodes of TB were common, all of which have been shown to exacerbate the toxicity profile of second-line drugs. However, the absence of comprehensive comorbidity data limits a more robust stratified analysis. Future work should prioritize detailed reporting of baseline comorbidities, including nutritional status, HIV status, and chronic conditions, to better inform both risk stratification and mitigation strategies, such as routine laboratory monitoring or early regimen adjustment.

The findings of this study have important clinical implications. The high incidence of definite ADRs, particularly hepatotoxicity and anemia, necessitates regular liver function tests and complete blood counts during TB treatment. The predominance of moderate ADRs highlights the need for routine monitoring and early intervention to prevent progression to severe ADRs.

## 5. Conclusions

Our study showed that 66.66% of people with DR-TB reported ADRs. This underscores the multifaceted nature of ADRs in pediatric TB treatment, influenced by age, weight, lung cavitation, and comorbidities. Our findings advocate for personalized treatment approaches, incorporating nutritional support, comprehensive comorbidity management, and vigilant monitoring to mitigate ADRs and improve treatment outcomes. Further research is warranted to explore the underlying mechanisms of ADR variability and to develop strategies for optimizing TB care in diverse patient populations.

## Figures and Tables

**Table 1:** Comparison of clinical and demographic characteristics of participants with and without ADRs.

Characteristics	Total (*n* = 450)	ADRs (*n* = 300)	No ADRs (*n* = 150)	*p*-Value
Gender				0.6
Male	217 (48.2)	147 (49.0)	70 (46.6)	
Female	233 (51.8)	153 (51.0)	80 (53.4)	
Age				0.001
0–4 years	21 (4.7)	16 (5.3)	5 (3.3)	
5–14 years	291 (64.7)	199 (66.3)	92 (61.3)	
15–16 years	138 (30.7)	85 (28.3)	53 (35.4)	
Weight				0.002
Underweight	346 (76.9)	214 (71.3)	132 (88.0)	
Normal weight	104 (23.1)	86 (28.7)	18 (12.0)	
Residence				0.4
Urban	330 (73.3)	224 (74.7)	106 (70.7)	
Rural	120 (26.7)	76 (25.3)	44 (29.3)	
Case Registration				0.002
New Case	152 (33.8)	99 (33.0)	53 (35.3)	
Retreated Case	298 (66.2)	201 (67.0)	97 (64.7)	
Type of DR-TB				0.2
MDR-TB	338 (75.1)	223 (74.3)	77 (25.7)	
MTB Rifampicin resistance	112 (24.9)	115 (76.7)	35 (23.3)	
Lungs Cavitation				0.001
Unilateral cavitation	138 (30.7)	92 (30.7)	208 (69.3)	
Bilateral cavitation	312 (69.3)	46 (30.7)	104 (69.3)	
Comorbidity				0.005
Yes	269 (59.8)	188 (62.7)	112 (37.3)	
No	181 (40.2)	81 (54.0)	69 (46.0)	

*p*-value was obtained using the Chi-square and Mann–Whitney U test.

**Table 2:** Treatment outcomes among drug-resistant participants.

Characteristics	*n* (%)
Cured	336 (74.7)
Treatment completed	20 (4.4)
Treatment failure	20 (4.4)
Loss to follow up	25 (5.6)
Died	24 (5.3)
Not evaluated	6 (1.3)
Shifted to Long-term regimen	19 (4.2)

**Table 3:** Types, incidence, and onset time of ADRs of ATT.

ADRs	Onset Time in Days Median (IQR)	Total (%)
Nausea and vomiting	22 (06–34)	55 (18.6)
Abdominal pain	62 (24–98)	47 (15.6)
Anorexia	25 (16–28)	10 (3.3)
Arthralgia	26 (16–52)	3 (1%)
Hepatotoxicity	44 (13–72)	41 (13.6%)
Allergy	12 (06–21)	31 (10.3%)
Anemia	15 (06–31)	113 (37.6%)

**Table 4:** Causality assessment of ADRs of ATT in children using the Naranjo causality scale.

Type of ADRs	Doubtful *n* (%)	Possible *n* (%)	Probable *n* (%)	Definite *n* (%)
Nausea/vomiting	0 (0.0)	8 (14.54)	17 (30.9)	30 (54.6)
Abdominal pain	3 (6.3)	23 (48.9)	6 (12.9)	15 (31.9)
Anorexia	1 (10)	4 (40)	2 (20)	3 (30)
Arthralgia	0 (0.0)	0 (0.0)	2 (66.66)	1 (33.34)
Hepatotoxicity	1 (2.3)	7 (17.6)	14 (32.8)	19 (47.3)
Allergy	5 (16.1)	3 (9.6)	2 (6.4)	21 (67.9)
Anemia	2 (1.7)	24 (21.3)	32 (28.3)	55 (48.7)

**Table 5:** Assessment of the severity of ADRs of ATT in children with the Hartwig severity assessment scale.

Type of ADRs	Number of ADRs (%)
Mild	92 (30.7)
Moderate	141 (47)
Severe	67 (22.3)

**Table 6:** Univariate analysis of risk factors for ADRs of ATT in children.

Characteristics	Total (*n* = 450)	ADRs (*n* = 300)	No ADRs (*n* = 150)	OR	*p*-Value
Gender					
Male	217 (48.2)	147 (49.0)	70		1
Female	233 (51.8)	153 (51.0)	80	0.9 (0.6–1.3)	0.64
Age					
0–4 years	21 (4.7)	16 (5.3)	5 (3.3)		1
5–14 years	291 (64.7)	199 (66.3)	92 (61.3)	1.3 (1.1–1.9)	0.05
15–16 years	138 (30.7)	85 (28.3)	53 (35.4)	0.3 (0.1–0.6)	0.001
Weight					
Underweight	346 (76.9)	214 (71.3)	132 (88.0)		1
Normal weight	104 (23.1)	86 (28.7)	18 (12.0)	0.3 (0.1–0.5)	<0.001
Residence					
Urban	330 (73.3)	224 (74.7)	106 (70.7)		1
Rural	120 (26.7)	76 (25.3)	44 (29.3)	1.2 (0.7–1.8)	0.3
Case Registration					
New Case	152 (33.8)	99 (33.0)	53 (35.3)		
Retreated Case	298 (66.2)	201 (67.0)	97 (64.7)	0.6 (0.5–1.3)	0.6
Type of DR-TB					
MDR-TB	338 (75.1)	223 (74.3)	77 (25.7)		1
MDR Rifampicin resistance	112 (24.9)	115 (76.7)	35 (23.3)	0.8 (0.5–1.3)	0.5
Lungs Cavitation					
Unilateral cavitation	138 (30.7)	92 (30.7)	208 (69.3)		1
Bilateral cavitation	312 (69.3)	46 (30.7)	104 (69.3)	0.2 (0.5–0.9)	0.005
Comorbidity					
Yes	269 (59.8)	188 (62.7)	112 (37.3)		1
No	181 (40.2)	81 (54.0)	69 (46.0)	0.3 (0.1–0.9)	<0.001

**Table 7:** Multivariate analysis of risk factors for ADRs of ATT in participants.

Variables	Β	AOR (95% CI)	*p*-Value
5–14 years	0.41	0.3 (0.1–0.5)	<0.001
15–16 years	0.82	1.5 (0.5–4.3)	0.2
Normal weight	1.14	1.1 (2.0–1.9)	<0.001
Bilateral cavitation	0.22	1.3 (1.2–5.2)	0.1
Comorbidity	0.55	0.5 (0.1–0.8)	<0.001

## Data Availability

The data will be made available by the authors upon request.
